# Can Ethnic Background Differences in Children’s Body Composition Be Explained by Differences in Energy Balance-Related Behaviors? A Mediation Analysis within the Energy-Project

**DOI:** 10.1371/journal.pone.0071848

**Published:** 2013-08-15

**Authors:** Juan Miguel Fernández-Alvira, Saskia J. te Velde, David Jiménez-Pavón, Yannis Manios, Amika Singh, Luis A. Moreno, Johannes Brug

**Affiliations:** 1 EMGO Institute for Health and Care Research and the Department of Epidemiology and Biostatistics, VU University Medical Center, Amsterdam, the Netherlands; 2 GENUD (Growth, Exercise, Nutrition and Development) Research Group), Faculty of Health Sciences, University of Zaragoza, Zaragoza, Spain; 3 Department of Nutrition and Dietetics, Harokopio University, Athens, Greece; Sapienza, University, Italy

## Abstract

**Background:**

In affluent countries, children from non-native ethnicity have in general less favourable body composition indicators and energy balance-related behaviors (EBRBs) than children from native ethnicity. However, differences between countries have been reported.

**Methodology/Principal Findings:**

A school-based survey among 10–12 years old children was conducted in seven European countries with a standardized protocol. Weight, height and waist circumference were measured; engagement in EBRBs was self-reported. For those countries with significant ethnic differences in body composition (Greece and the Netherlands), multilevel mediation analyses were conducted, to test the mediating effect of the EBRBs in the association between ethnic background and body composition indicators. Analyses were adjusted for gender and age, and for parental education in a later step. Partial mediation was found for sugared drinks intake and sleep duration in the Greek sample, and breakfast in the Dutch sample. A suppression effect was found for engagement in sports activites in the Greek sample.

**Conclusions/Significance:**

Ethnic differences in children’s body composition were partially mediated by differences in breakfast skipping in the Netherlands and sugared drinks intake, sports participation and sleep duration in Greece.

## Introduction

Childhood overweight and obesity are a major public health issue in many parts of the world [1]. The obesity epidemic does not affect all segments of the population equally. Differences according to socio-economic position or level of education have been well documented [2], [3], also for school-aged children [4], [5]. Recent American [6], [7] and European [8]–[10] studies show ethnic inequalities in childhood overweight and obesity prevalence [11], [6], [12]–[14], [7], [15], [16] and energy balance-related behaviors (EBRBs) [7], [17]–[21] with ethnic minorities showing higher prevalence rates. A recent publication [22] on the results of the cross-sectional study of the “EuropeaN Energy balance Research to prevent excessive weight Gain among Youth” (ENERGY)-project [23] also showed that children of native ethnicity of the country of residence had, in general, more favorable weight status indicators and EBRBs than children of non-native ethnicity across seven countries in Europe. However, interesting differences were observed between countries. Most importantly, the largest differences between native and non-native children were found in the Netherlands. In the Netherlands, the prevalence of overweight among non-native children was almost twice the prevalence among native Dutch children (26% vs. 15%). Conversely, an opposite pattern (e.g. significantly lower Body Mass Index (BMI) and waist circumference (WC) in non-native children compared to native children) was observed in the Greek sample.

**Figure 1:**
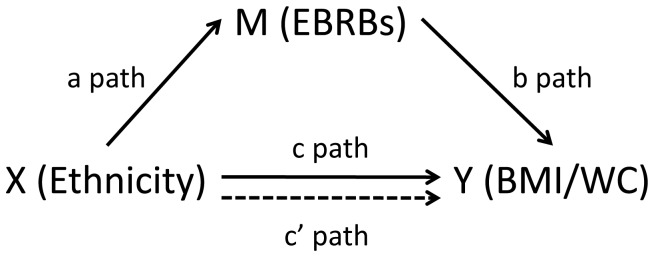
Mediation mode. X: predictor variable; Y: outcome variable; M: mediator variable; a path: association between predictor (X) and potential mediator (M); b path: association between potential mediator (M) and outcome variable (Y); c: overall association between predictor variable (X) and outcome variable (Y); c’: direct effect (unmediated) of predictor variable (X) on outcome variable (Y).

In order to understand what behaviors may drive and explain these inequalities, it is of special interest to study the underlying mechanisms in the countries where we found differences between native and non-native groups (e.g. Greece and the Netherlands). Understanding if and how different EBRBS may be associated with ethnic differences in body composition may help to tailor preventive intervention initiatives aiming at reducing these ethnic inequalities. These underlying mechanisms can be assessed by mediation analyses [24] (see figure 1).

The current study therefore aims to assess if specific EBRBs mediate the association between ethnicity and body composition indicators found in the Dutch and Greek subsamples of the ENERGY cross-sectional study [22]. More specifically and following the stepwise approach for mediation analyses, the aims are: 1) to assess the total associations of ethnic background (see figure 1) with two different body composition outcomes: BMI and WC (c path); 2) to assess the associations of ethnic background with EBRBs as potential mediating variables (a path); and 3) to assess the mediated pathways of EBRBs on BMI and WC (a*b).

## Methods

### Study Population and Design

Data were obtained from the cross-sectional study of the ENERGY project [23]. This cross-sectional study was carried out between March and July 2010 in Belgium, Greece, Hungary, the Netherlands, Norway, Slovenia and Spain, among pupils in the final years of primary education (aged 10–12) [25]. Samples were national representative both in Greece and in the Netherlands (along with Hungary and Slovenia). The aim of the survey was to provide up to date information on the prevalence of overweight and obesity, on the most important EBRBs and their socio-demographic, personal and family- and school-environmental correlates. Descriptions of the rationale and design of the entire ENERGY project [23] and the procedures and methodology of the ENERGY school-based survey are published elsewhere [25]. As mentioned before, the prevalence rates of overweight, obesity and engagement in different EBRBs, as well as differences in these prevalences according to parental education and ethnicity, have been published before [5], [22]. The study was performed following the ethical guidelines of the Declaration of Helsinki 1964 (revision of Edinburgh 2000), the Good Clinical Practice, and the legislation about clinical research in humans in each of the participating countries. All participating countries obtained ethical clearance from the relevant ethical committees and ministries. In Greece the survey was approved by the Bioethics Committee of Harokopio University and in the Netherlands the survey was approved by the Medical ethics Committee of the VU University Medical Center; In addition, research permission was obtained, if necessary from local school authorities. A school recruitment letter was sent to the headmaster or principal of the sampled schools. After school’s agreement, parents received a letter explaining the study purpose and were asked for written consent for their child’s and own participation.

### Measures

Measurements were conducted following standardized protocols. The children and their parents were asked to complete questionnaires assessing obesity related dietary, physical activity and sedentary behaviors, as well as potential determinants of engaging in these behaviors. Test-retest reliability and construct validity were tested by administrating the questionnaire twice with a week interval among 720 children across the participating countries. The intraclass coefficients and percentage agreement was good to excellent for 77% of items and construct validity was moderate to excellent for 73% of items [26]. Detailed information regarding the procedures, staff training and questionnaires development [25] and test-retest reliability and construct validity of the questionnaires are published elsewhere [26], [27].

#### Anthropometric measurements

Body height, weight and WC were measured by trained research assistants. Children were measured in light clothing without shoes. Body height was measured with a SECA Leicester Portable stadiometer (to the nearest 0.1 cm). Weight was measured with a calibrated electronic scale SECA 861 (to the nearest 0.1 kg), and WC with a SECA 201 measuring band (to the nearest 0.1 cm). Two readings of each measurement were obtained. A third measurement was taken if the two readings differed more than 1%. BMI [calculated as body weight (kg) divided by the height (m) squared (kg/m2)].

#### Ethnic background

Regarding ethnic background we made a distinction between children of immigrant origin (non- native) and children of native origin in the country of administration based on the language spoken at home and [28]. We assessed in the child questionnaire which language was mostly spoken in the home environment of the child. The answering categories were tailored to the different countries, including the official language or languages of the specific country or region, the native languages of the largest ethnic minorities, and a category ‘other’. We created a dichotomous variable distinguishing those children for whom the official language of the country of administration was mainly spoken at home (e.g. Greek in Greece; Dutch in the Netherlands, native) from those who reported another language as the main language at home (non-native).

#### Children’s EBRBs

Sugared drinks consumption, breakfast consumption, active transport, sports participation, TV viewing and computer use were assessed by the child self-report, while sleep duration was reported by the parents. These specific behaviors were selected based on a review study conducted within the ENERGY project of earlier prospective studies on EBRBs in association with overweight indicators [29].

#### Dietary behaviors

Intakes of soft drinks and fruit juices were assessed with two food frequency questions. Children answered how many days per week they drank soft drinks (ICC_test-retest_ = 0.71) and fruit juices (ICC_test-retest_ = 0.64) answering on a seven-point scale from never to more than once every day. Afterwards they were asked to indicate how much they drank by reporting the number of glasses or small bottles (e.g. 250 ml), cans (i.e. 330 ml) and large bottles (i.e.500 ml) for soft drinks, or glasses/small cartons (i.e.250 ml) and regular cartons (330 ml) for fruit juices. The questionnaire included pictures of the serving sizes. These items showed moderate to good reliability (ICC_test-retest_ = 0.53–0.71). Mean intake in ml per day was calculated from these two questions. In addition, children were asked to fill in how much of the beverages they had consumed on the day before, following the same classification. For the purposes of this analysis, liters/day of sugar-containing drinks (soft drinks+fruit juices) were taken into account. Breakfast consumption was assessed with two food frequency questions. Children answered how many days they usually eat breakfast during school days (ICC_test-retest_ = 0.73) and in the weekend (ICC_test-retest_ = 0.52). Breakfast frequency per week was calculated by adding up the answers of the two previous questions. For the purposes of this analysis, the frequency was recoded into 0 (had breakfast 0–6 times week) and 1 (had breakfast 7 days week).

#### Physical activity

Active transportation to school was assessed by two questions about how many days per week the child cycled and/or walked to school (ICC_test-retest_  = 0.94 and 0.91), ranging from never to 5 days/week, and two questions on the duration of biking or walking to school, with 4 answer categories ranging from 1–5 minutes to more than 15 minutes (ICC_test-retest_  = 0.81 and 0.70). Total active transportation time per week was calculated by adding up total bike and walk times and multiplying the number of days with the mean time of the answering category times 2. Organized sports participation was assessed with specific questions about how many hours per week children participated in one or two sports (ICC_test-retest_ = 0.74 and 1.00). Based on the answers, average time of sports participation per week was calculated. Finally, minutes/day of active transportation and min/day of sports participation were included in the analysis.

#### Screen behaviors

Screen time (i.e. TV and computer time) was assessed separately for weekdays and weekend days by two questions about time spent watching TV (including video and DVD) (ICC_test-retest_ = 0.67 and 0.68) and computer activities (ICC_test-retest_ = 0.67 and 0.67). Mean TV, computer and total screen time per day were calculated. For the analysis, total hours/day of screen time were taken into account.

*Sleep duration.* Child’s sleep duration reported by the parents included the number of hours the child sleeps per night on average, separately for weekdays (ICC_test-retest_  = 0.81) and weekend days (ICC_test-retest_  = 0.78). Mean number of hours of sleep per day was calculated.

#### Parental educational level

As an indicator of socio-economic status, we asked the parents to report their own level of education, as well as the level of education of the other parent/caregiver. Because educational systems differ considerably across Europe, the number of years of formal education was used as the indicator for level of education. Parental education was categorized as being high (i.e. at least one parent more than 14 years of education) or low (i.e. both parents less than 14 years of education), which approximately distinguishes families with at least one caregiver who has completed medium or higher vocational, college or university training from other families [30].

### Statistical Analysis

Descriptive statistics and unadjusted analyses (MANOVA and Chi square tests) were performed using the Statistical Package for the Social Sciences (SPSS) (Version 20.0, SPSS Inc., Chicago, IL). Logistic regression analyses were conducted to check whether non-response was associated with the ethnicity indicator and other basic characteristics (e.g. gender, BMI).

#### Mediation analysis

To assess whether the associations between ethnicity and body composition outcomes were mediated by the EBRBs, mediation analyses were conducted following a stepwise approach. Because children were nested within schools and some of the ICCs were higher than 0.05, multilevel linear regression analyses were performed to assess associations between the ethnicity indicator, body composition and potential mediators using Mplus software (Version 6.11, Muthén & Muthén). Steps 2–4 were conducted in Mplus [31] using the INDIRECT command and adjusting for the clustered design (children nested in schools, using the CLUSTER command).

Step 1: although a significant overall association (c-path) is not a prerequisite for conducting mediation analyses, in this study we only assessed potential underlying pathways if a significant difference in body composition outcomes between native and non-native children was observed. These analyses were conducted before [22] and based on these previous observations, we included only the Greek and Dutch subsamples, as the association between language spoken at home and body composition indicators was not significant in the rest of the participating countries [22].

Step 2: to qualify as a mediator, the presumed mediator (M) has to be associated with the predictor variable (a-path) [32]. This was assessed by regressing the potential mediators (M, the EBRBs) on the ethnicity indicator (X) as an independent dichotomous variable (non-native  = 0, native  = 1).

Step 3: to qualify as a mediator, the presumed mediator (M) has also to be associated with the outcome (Y, b-path). This was assessed by regressing the outcome variables (body composition indicators) on the mediators (EBRBs).

Step 4: to estimate the mediated effect (a*b) the product-of-coefficients test proposed by MacKinnon was performed [33] (see Figure 1). The mediated proportions were calculated as the mediation effect divided by the total effect (path c) ([a_i_*b_i_]/c). Total effects were estimated by regression models without the potential mediators. Standard errors were calculated and used to construct the 95% confidence intervals (CI) for the direct and total effects.

Step 5: finally, direct associations between the ethnicity indicator and body composition indicators were assessed including the mediators in the models.

All steps were adjusted for age and gender in a first step, and also including parental education in a second step. Due to the low parental participation rate in the Dutch sample (less than 50%) models adjusted for parental education will be presented only for the Greek sample.

## Results

### Participant Characteristics

The total sample comprised 2002 children (Table 1). The large majority of children were of native ethnicity. Non-native children presented significantly higher BMI and WC in the Dutch sample and significantly lower BMI and WC in the Greek sample. Non-native groups showed a significantly higher percentage of lower educated parents in both Greek and Dutch subsamples (Table 1).

**Table 1:** Characteristics of participants.

	Non-native	Native	P value*
**Greece**			
N (%)	108 (10.0)	975 (90.0)	
Age (mean, SD)	11.4	11.3	0.190
Gender (% males)	45.4	46.1	0.018
Parental education level (%)			
Low	71.2	46.3	<0.001
High	28.8	53.7	
BMI (mean, SD)	19.5 (3.7)	20.5 (3.8)	0.009
WC (mean, SD)	68.1 (9.6)	70.8 (9.8)	0.008
**Netherlands**			
N	75 (8.2)	844 (91.8)	
Age (mean, SD)	11.9	11.7	0.055
Gender (% males)	50.7	49.5	0.036
Parental education level (%)			
Low	66.7	20.8	0.001
High	33.3	79.2	
BMI (mean, SD)	19.8 (3.8)	18.3 (3.0)	<0.001
WC (mean,SD)	66.3 (8.9)	63.0 (7.3)	<0.001

* Student’s t-test (age, BMI, weight) and Pearson Chi-Square (% males, % parental education).

Parental response was much lower in the Netherlands (44%) compared with Greece (81%). Logistic regression analyses showed that non-response by parents was associated with language spoken at home in both samples, and also with age and BMI in the Dutch sample. Parents of older (Dutch sample OR = 0.60; CI = 0.50–0.72), heavier children (Dutch sample OR = 0.85; CI = 0.85–0.94) and non-natives (Greek sample OR = 0.40; CI = 0.26–0.63) (Dutch sample OR = 0.28; CI = 0.15,0.54) were less likely to complete the parental questionnaire.

### Energy Balance-Related Behaviors (EBRBs)

In the Greek sample sugared drink intake and sleep duration were significantly higher, and sports participation significantly lower among non-native children (Table 2). Regarding the Dutch sample, non-native children reported significantly higher breakfast skipping.

**Table 2:** Means and SD for dietary, physical activity and sedentary behaviors in the Greek and Dutch samples, for native and non-native children based on language spoken at home.

		Non-native (n = 108)	Native (n = 974)
**Greece**	Sugared drinks (L/day)	0.514±0,545	0.359±0,326***
	Skipped breakfast ≥1 week (%)	50.0	46.8
	Active transport (min/day)	9.6±7.7	8.8±7.7
	Sports participation (min/day)	14.8±19.9∧∧∧	23.9±20.7
	Screen time (hours/day)	3.48±1.49	3.23±1.59
	Sleeping habits (hours/day)	9.0±1.1	8.7±0.8**
		**Non-native (n = 64)**	**Native (n = 769)**
**Netherlands**	Sugared drinks (L/day)	1.151±1.094	0.993±0.733
	Skipped breakfast ≥1 week (%)	37.5	21.8**
	Active transport (min/day)	12.4±10.2	12.0±9.3
	Sports participation (min/day)	25.8±21.1	30.1±21.5
	Screen time (hours/day)	3.83±1.95	3.35±1.80
	Sleeping habits (hours/day)	9.3±0.9	9.6±0.7

* P<0.05;

** P<0.01;

*** P<0.001 native significantly lower than non-native, adjusted for age and gender.

∧ P<0.05;

∧∧ P<0.01;

∧∧∧ P<0.001 non-native significantly lower than native, adusted for age and gender.

Taking into account the described results, those EBRBs showing significant differences between native and non-native children were selected to test their potential mediating effect on the significant associations between the ethnicity indicator and body composition indicators (e.g. sugared drinks, sports participation and sleeping habits in the Greek sample; skipping breakfast in the Dutch sample).

#### Step 1: ethnic background and body composition indicators (c-path)

The results showed that language spoken at home was significantly associated with BMI and WC in Greek and Dutch samples, but in different directions: Greek native children had higher BMI and WC compared to non-natives (Table 3), while Dutch native children showed lower BMI and WC (Table 4).

**Table 3:** Regresion coefficients (B) and 95% confidence intervals (95% CI) as results from the mediation analyses in the association between language spoken at home (non-native = 0, native = 1) and BMI (kg/m^2^)/WC (cm) in Greek simple.

BMI	Total association	Languageeffect on	Single mediatoreffect on	Indirect effect	Direct effect	Percentage
Mediator:	(path c)	mediator (path a)	BMI (path b)	(a*b)	(path c’)	mediated
	B (95% CI)	B (95% CI)	B (95% CI)	B (95% CI)	B (95% CI)	(a*b/c)
Sugared drinks (L/day)*(n = 1081)	**1.04 (0.37; 1.72)**	**−0.15** **(−0.28; −0.03)**	**−1.15** **(−1.81; −0.49)**	**0.176** **(0.006; 0.347)**	**0.87** **(0.21; 1.52)**	16.9
Sports (Min/day)*(n = 1081)	**1.03 (0.36; 1.70)**	**9.07** **(5.58; 12.56)**	−0.007(−0.019; 0.004)	−0.067(−0.174; 0.040)	**1.10** **(0.41; 1.78)**	–
Sleep (Hours/day)*(n = 1078)	**1.04 (0.36; 1.71)**	**−0.30** **(−0.52; −0.08)**	**−0.56** **(−0.84; −0.29)**	**0.169** **(0.009; 0.329)**	**0.87** **(0.22; 1.52)**	16.2
Sugared drinks (L/day)†(n = 890)	**1.11 (0.12; 2.10)**	**−0.08** **(−0.17; 0.00)**	**−1.13** **(−2.02; −0.24)**	0.093(−0.003; 0.219)	**1.02** **(0.05; 1.99)**	–
Sports (Min/day)†(n = 890)	**1.09 (0.11; 2.07)**	**9.91** **(5.53; 14.30)**	−0.005(−0.016; 0.007)	−0.049(−0.163; 0.065)	**1.14** **(0.16; 2.12)**	–
Sleep (Hours/day)†(n = 890)	**1.10 (0.12; 2.08)**	**−0.18** **(−0.19; −0.03)**	**−0.49** **(−0.81; −0.17)**	0.087(−0.034; 0.207)	**1.01** **(0.05; 1.98)**	–
**WC**	**Total association**	**Language** **effect on**	**Single mediator** **effect on**	**Indirect effect**	**Direct effect**	**Percentage**
**Mediator:**	**(path c)**	**mediator** **(path a)**	**WC (path b)**	**(a** * **b)**	**(path c’)**	**mediated**
	**B (95% CI)**	**B (95% CI)**	**B (95% CI)**	**B (95% CI)**	**B (95% CI)**	**(a** * **b/c)**
Sugared drinks (L/day) *(n = 1081)	**2.83 (0.99; 4.67)**	**−0.15** **(−0.28; −0.03)**	**−2.53** **(−4.36; −0.71)**	**0.390** **(0.039; 0.740)**	**2.44** **(0.68; 4.19)**	13.8
Sports (Min/day)*(n = 1081)	**2.80 (0.97; 4.63)**	**9.07** **(5.58; 12.55)**	**−0.031** **(−0.056; −0.007)**	**−0.285** **(−0.544; −0.027)**	**3.09** **(1.26; 4.92)**	−10.1
Sleep duration (Hours/day)*(n = 1078)	**2.81 (0.98; 4.65)**	**−0.30** **(−0.52; −0.08)**	**−1.11** **(−1.82; −0.40)**	0.331(−0.024; 0.686)	**2.48** **(0.67; 4.30)**	–
Sugared drinks (L/day)†(n = 890)	**2.78 (0.19; 5.37)**	−0.08(−0.17; 0.00)	**−2.59** **(−5.11; −0.07)**	0.213(−0.104; 0.529)	**2.57** **(0.06; 5.07)**	–
Sports (Min/day)†(n = 890)	**2.73 (0.16; 5.30)**	**9.89** **(5.50; 14.28)**	−0.025(−0.052; 0.002)	−0.247(−0.524; 0.031)	**2.98** **(0.44; 5.52)**	–
Sleep duration (Hours/day)†(n = 890)	**2.75 (0.18; 5.32)**	−0.18(−0.39; 0.03)	**−0.89** **(−1.70; −0.07)**	0.157(−0.096; 0.411)	**2.59** **(0.07; 5.12)**	–

* Models adjusted for age and gender.

† Models adjusted for age, gender and parental education level.

path a, b,c c’refer to the paths depicted in Figure 1.

**Statistically significant associations are shown in bold.**

**Table 4:** Regresion coefficients (B) and 95% confidence intervals (95% CI) as results from the mediation analyses in the association between language spoken at home (non-native = 0, native = 1) and BMI (kg/m^2^)/WC (cm) in Dutch simple.

BMI	Total association	Language effect on	Single mediatoreffect on	Indirect effect	Direct effect	Percentage
Mediator:	(path c)	mediator (path a)	BMI (path b)	(a*b)	(path c’)	mediated
	B (95% CI)	B (95% CI)	B (95% CI)	B (95% CI)	B (95% CI)	(a*b/c)
Breakfast*(n = 911)	**−1.30** **(−1.79; −0.81)**	**2.16 (1.30; 3.61)** **	**−0.67** **(−0.99; −0.34)**	**−0.309 (−0.600;** **−0.018)**	**−0.99 (−1.48;** **−0.50)**	23.7
**WC**	**Total association**	**Language effect on**	**Single mediator** **effect on**	**Indirect effect**	**Direct effect**	**Percentage**
**Mediator:**	**(path c)**	**mediator (path a)**	**WC (path b)**	**(a** * **b)**	**(path c’)**	**mediated**
	**B (95% CI)**	**B (95% CI)**	**B (95% CI)**	**B (95% CI)**	**B (95% CI)**	**(a** * **b/c)**
Breakfast*(n = 911)	**−2.76** **(−4.36; −1.17)**	**2.16 (1.30; 3.61)** **	**−1.30** **(−2.15; −0.45)**	**−0.603 (−1.209;** **−0.094)**	**−2.16 (−3.69;** **−0.62)**	21.8

* Models adjusted for age and gender.

** Logistic regression coefficients.

path a, b,c c’refer to the paths depicted in Figure 1.

**Statistically significant associations are shown in bold.**

#### Step 2: Associations between ethnic background and EBRBs (a-path)

As described in tables 3 and 4, significant associations with language spoken at home were found for consumption of sugared drinks, sports participation, and sleep duration in the Greek sample, while significant associations were found for breakfast in the Dutch sample. Sugared drinks intake and sleep duration were lower in the native Greek group. The odds of having breakfast everyday were significantly higher in the native Dutch children.

#### Step 3: Associations between EBRBs and children’s body composition (path-b)

In the Greek sample consumption of sugared drinks and sleep duration were inversely associated with both BMI and WC. Sports participation only showed significant inverse association with WC and only in the model not adjusted for parental education. In the Dutch sample, having breakfast everyday was inversely associated with BMI and WC.

#### Step 4: Mediation effects (a*b)

Indirect effects are shown in the 4^th^ column of tables 3 and 4. Mediation effects in the Greek sample were statistically significant for consumption of sugared drinks in both models (BMI and WC). The mediated proportions varied, with higher proportion for the BMI model (16.9%) compared to the WC model (13.8%). Mediation effect was also significant for sleep duration in the BMI model (16.2%). Sports participation had a suppressive effect on the relationship between language spoken at home and WC (−10.1%), due to the opposite directions of the direct and indirect associations, indicating an increase of the association between the predictor (language) and the outcome (WC). After adjusting for parental education, none of the variables showed significant mediation effect (table 3).

Regarding the Dutch sample (table 4), breakfast significantly mediated the association between language spoken at home and BMI/WC (23.7% and 21.8% respectively).

#### Step 5: Direct association (path-c’)

As presented in tables 3 and 4, the direct significant associations between ethnicity and body composition indicators remained significant after the inclusion of the presumed mediators in the models.

## Discussion

The present study explored if specific EBRBs could explain differences in body composition according to ethnic background, in two countries where ethnic differences in overweight status were present. In the Netherlands non-native children were more likely and in Greece non-native children were less likely to be overweight than native children (25). Partial mediation in the associations between ethnic background and children’s body composition was found only for consumption of sugared drink and sleep duration in the Greek sample, and breakfast in the Dutch sample. A suppression effect was found for sports in the Greek sample, indicating that the magnitude of the relationship between ethnicity and WC became larger after the inclusion of the mediator in the model. After adjusting the mediation models for parental education in the Greek sample, no mediation was found.

The results suggest that differences in children’s body composition between Dutch native and non-native children may be partially due to the higher proportion of breakfast skipping in non-native children. This finding is consistent with previous studies in which skipping breakfast is associated with a less favorable body composition in both children and adolescents [34]. Previous reports also showed higher percentages of breakfast skipping among non-native children [7], [35], [16] but did not assess mediation effects of breakfast skipping on the association between ethnicity and children’s body composition.

Unlike in the Netherlands, and most other reports on ethnic differences [36], [7], [16], [10], Greek native children had higher BMI and WC compared to non-natives. Greek native children have the highest BMIs and WCs in Europe [5], and prevalence of overweight and obesity in Greek children is amongst the highest of the world [37]. Compared to a population that is amongst the most overweight in the world, it is very likely that children with other ethnic backgrounds have lower overweight rates. Furthermore, many immigrants in Greece are from the most deprived countries in Europe, e.g. Albania and Romania, which may also account for their somewhat lower rates of overweight and obesity compared to native Greek children. However, non-native children in Greece still had higher BMI and prevalence of overweigh and obesity than children in most other countries in Europe, suggesting that Greece has certain specific obesogenic characteristics.

The mediation analyses indicate that differences regarding sugared drink consumption, sleep duration and sports participation partially explain the existing body composition differences according to ethnicity in Greece. In agreement with the literature [38]–[40], sleep duration was inversely associated with body composition indicators, and was also found to be shorter in native Greek children, partially explaining the higher BMI and WC in native Greeks. Also in agreement with previous studies, native children reported lower sugared drinks intake [12], but unexpectedly and in contrast with other studies [41], [42] sugared drinks consumption was inversely associated with body composition in this Greek sample. These results may reflect actual lower intake of sugared drinks in overweight children in this country –suggesting that other behaviors are of key importance in Greece, or a higher impact of underreporting in this group [43], [44].

A suppression effect was found for sports participation in the Greek sample. As expected, sports participation was inversely associated with WC, but native children reported higher sports participation, even though they presented higher WC values. It has to be kept in mind that the information regarding sports reflects the engagement in structured sports activities, and not total physical activity. It is possible that non-native children may be less likely to get involved in sports clubs, for example because of financial barriers, but do engage in more daily physical activities even achieving a total amount of energy expenditure higher than the native Greek children.

To our knowledge, this is one of the first studies exploring the pathways between ethnicity, several EBRBs of special relevance in school-aged children, and children’s body composition by formally applying mediation analysis. The proportion of the associations explained by the identified mediators was relatively low, and a direct association between ethnicity and body composition remained statistically significant. That the proportion mediated was relatively low is not surprising as we only included one mediator in the models and it is very likely that many other factors (not measured in the ENERGY-study) can explain the observed ethnic differences. Furthermore, with large sample sizes it is very hard to find complete mediation. Nevertheless, it indicates that other relevant mediators, e.g. other health behaviors, may be of additional importance. Therefore, future studies should take into account more EBRBs in order to better explain the ethnic differences in body composition.

The mediated effects became non-significant after adjusting for parental education in the Greek sample. This finding may reflect the close relationship between ethnicity and parental educational and suggests an important role of the later in the associations between ethnicity, EBRBs and children’s body composition. However, the sample size was substantially smaller in the models including parental education, due to the fact that this was reported in the parent questionnaire, which unfortunately had a lower response rate and obviously reduced statistical power. Additionally, further logistic regression analyses showed that parents from non-native children were less likely to complete the parental questionnaire (Greek sample OR = 0.40; CI = 0.26–0.63). This might imply a bias in the results, probably leading to weaker associations due to less variation and may explain the non-significant findings after adjusting the models by parental education.

Using the present analyses, we were not able to completely explain ethnic differences in body composition by the included EBRBs. The unexplained differences may be a result of the interaction with other variables (e.g. socioeconomic determinants, cultural background, biological factors) that determine the existing ethnic differences in children’s body composition. It is also noteworthy that the EBRBs found to be mediating the ethnic differences in body composition are different for each of the assessed countries. This finding reflects the differences between countries and the need for tailored overweight prevention interventions.

A number of limitations should be taken into account. First of all, the results presented are based on cross-sectional data, and thus no causality can be established. Second, data on dietary, physical activity and sedentary behaviors were assessed by self-reports, and may be biased. Nevertheless, the measures showed good test-retest reliability and construct validity [26], [27]. Additionally, the differences in response rates at school level were important, and may have an impact on the external validity of the findings. The response rates among schools and parents were lower in the Netherlands, underlining experiences with other Dutch school-based research programs over the last years. Missing data analysis revealed that parents of older and heavier children in the Dutch samples and non-native children in both samples were less likely to complete the parental questionnaire. Therefore, the results including adjustment for parental education should be interpreted carefully.

Strengths of the current study include the standardized data collection protocol across the different countries and the measured weight, height and WC data along with the inclusion of behaviors that have been widely associated with overweight and obesity in children and adolescents.

### Conclusion

Ethnic differences in children’s body composition were partially mediated by differences in breakfast skipping in the Netherlands and consumption of sugared drinks, sports participation and sleep duration in Greece. More studies are needed to disentangle which variables are able to further explain the ethnic differences in children’s body composition.
